# Assessing How Risk Communication Surveillance Prompts COVID-19 Vaccine Acceptance Among Internet Users by Applying the Situational Theory of Problem Solving: Cross-Sectional Study

**DOI:** 10.2196/43628

**Published:** 2023-07-26

**Authors:** Qiang Jin, Syed Hassan Raza, Muhammad Yousaf, Umer Zaman, Emenyeonu C Ogadimma, Amjad Ali Shah, Rachel Core, Aqdas Malik

**Affiliations:** 1 Intercultural Communication Research Center, Hebei University Baoding China; 2 Institute of Media and Communication Studies, Bahauddin Zakariya University Multan Pakistan; 3 Centre for Media and Communication Studies, University of Gujrat Gujrat Pakistan; 4 Endicott College of International Studies, Woosong University Daejeon Republic of Korea; 5 College of Communication, University of Sharjah Sharjah United Arab Emirates; 6 Sociology & Anthropology Department, Stetson University DeLand, FL United States; 7 Department of Information Systems, Sultan Qaboos University Muscat Oman

**Keywords:** COVID-19, vaccine safety, risk communication, digital interventions, health communication, Situational Theory of Problem Solving

## Abstract

**Background:**

The World Health Organization has recently raised concerns regarding the low number of people fully vaccinated against COVID-19. The low ratio of fully vaccinated people and the emergence of renewed infectious variants correspond to worsening public health. Global health managers have highlighted COVID-19 vaccine–related infodemics as a significant risk perception factor hindering mass vaccination campaigns.

**Objective:**

Given the ambiguous digital communication environment that has fostered infodemics, resource-limited nations struggle to boost public willingness to encourage people to fully vaccinate. Authorities have launched some risk communication–laden digital interventions in response to infodemics. However, the value of the risk communication strategies used to tackle infodemics needs to be evaluated. The current research using the tenets of the Situational Theory of Problem Solving is novel, as it explores the impending effects of risk communication strategies. The relationship between infodemic-induced risk perception of COVID-19 vaccine safety and risk communication actions to intensify willingness to be fully vaccinated was examined.

**Methods:**

This study used a cross-sectional research design vis-à-vis a nationally representative web-based survey. We collected data from 1946 internet users across Pakistan. Participants voluntarily participated in this research after completing the consent form and reading ethical permissions. Responses were received over 3 months, from May 2022 to July 2022.

**Results:**

The results delineated that infodemics positively affected risk perception. This realization pushed the public to engage in risky communicative actions through reliance on and searches for accurate information. Therefore, the prospect of managing infodemics through risk information exposure (eg, digital interventions) using the situational context could predict robust willingness to be fully vaccinated against COVID-19.

**Conclusions:**

These pioneering results offer strategic considerations for health authorities to effectively manage the descending spiral of optimal protection against COVID-19. This research concludes that the likelihood of managing infodemics using situational context through exposure to relevant information could improve one’s knowledge of forfending and selection, which can lead to robust protection against COVID-19. Hence, more situation-specific information about the underlying problem (ie, the selection of an appropriate vaccine) can be made accessible through several official digital sources to achieve a more active public health response.

## Introduction

The World Health Organization (WHO) reported 596,873,121 COVID-19 cases and 6,459,684 deaths as of August 26, 2022 [1]. The uptake of COVID-19 vaccination prevents severe disease [2]. Numerous studies have shown that COVID-19 vaccinations reduce respiratory problems, severe symptoms, hospitalization, and death [3]. COVID-19 vaccinations were initially administered internationally. However, many nations that promptly immunized a large proportion of their populations found that protective antibodies started to wane over time or were ineffective against COVID-19 variants [4]. To date, these variants have lingered, often infecting people on a large scale, which has resulted in mounting pressure on health care systems. Health officials suggested 4 shots in a 2-dose series or 2 single-dose series vaccines to be “fully vaccinated” against severe COVID-19 illness. As of August 23, 2022, 4.92 billion people (63.1%) have received COVID-19 vaccines [1]. On August 23, 2022, the WHO reported over 5 million new COVID-19 cases, and 13,914 deaths were reported a week prior. This prognosis alarmed resource-limited nations with health care concerns.

Despite its proven efficacy as a precautionary health care strategy, vaccine uptake rates worldwide among adults are a concern, particularly in resource-limited nations [5]. A large proportion of the population in the developing world remains unvaccinated or is not fully vaccinated. The reluctance to be fully vaccinated is one of the primary causes of the emergence of new variants [6]. According to the WHO, infodemics are the foremost reason for the unwillingness to vaccinate fully. Infodemic is defined as the overabundance of misinformation or confusing information across digital and physical environments during a pandemic that can potentially mislead the public [7] and shake trust in health authorities and public health responses [8]. Therefore, responding to the rapid spread of rumors and conspiracy theories in the form of infodemics related to the pandemic is particularly urgent to increase the awareness and efficacy of vaccines [9,10].

COVID-19 vaccine–related infodemics may influence vaccination decisions [11]. Receiving vaccine information from various sources does not automatically lead to vaccine acceptance or refusal [12]. Notably, health information sources, particularly health authorities, may shape individuals’ attitudes or beliefs about vaccination [13,14], which may affect vaccine uptake [2]. Despite a wealth of literature [15], some key gaps remain unexplored. First, few studies have examined the effects of multiple sources on COVID-19 vaccine attitudes and uptake [16,17]. COVID-19 research has focused on one health information source and yielded inconsistent results [6,18,19]. Most COVID-19 vaccination literature and information sources have focused on vaccine safety [20].

Little is known about how infodemics may influence negative vaccine safety perceptions [21]. The impact of digital interventions on infodemics and vaccination rates is unknown. Previous research suggests that public trust in vaccines and authorities determines safety-related health concerns [22]. Trust reduces safety concerns and vice versa. Thus, increased confidence in the COVID-19 vaccine brand or country of origin could improve brand safety [16]. Multifaceted infodemics, particularly those that are negative for vaccines and their countries of origin, can lower safety perception [23]. This phenomenon regulates perceptual evaluation and situational motivation [11,24]. Given the abundance of information regarding the pros and cons of several COVID-19 vaccines [17,20,22], it is worthwhile to delineate the novel mechanism involved in an emerging problem. These questions determine public willingness to get fully vaccinated against COVID-19 (WFVC): what factors influence COVID-19 vaccine choice? Can risk-communicative infodemic management improve the WFVC? This study answers these understudied questions about health information interventions in the context of the Situational Theory of Problem Solving (STOPS) and provides several managerial implications. Therefore, this study aimed to (1) predict the impact of infodemics on COVID-19 vaccine safety risk perception, (2) examine how risk perception affects individuals’ communication behavior in problem solving, and (3) evaluate the effectiveness of health authorities’ information management strategies using a health information–seeking approach.

## Methods

### Theoretical Framework and Hypotheses Development

#### The STOPS

The STOPS highlights perceptual and situational variables that demonstrate certain public perceptions that can further define communicative behaviors [25]. In the context of COVID-19, perception of the problem can emerge because of the potential side effects of a particular COVID-19 vaccine spread through infodemics (eg, spread through social media or personal networks). Nonetheless, selecting the safest vaccine brand for inoculation could be a solution for minimizing pandemic threats (ie, reduced problem recognition). By contrast, self-efficacy in choosing a particular vaccine brand can hinder recognition and prevent inoculation. Thus, constraint recognition incorporates people’s impressions of their inability to lessen the health risks and side effects of the COVID-19 vaccine due to perceived constraints. These difficulties stem from infodemics [17], disinformation [26], and conspiracy theories [27]. This can lower their situational motivation and other vaccination behavior.

The last perceptual variable is the extent to which one thinks that family and friends are susceptible to the problem [15,28] and the potential adverse effects of COVID-19 (ie, involvement recognition). However, the perceived safety of a COVID-19 vaccination brand determines its perceptual aspects. People may be less worried about vaccine safety if they think that a brand is safe. The STOPS stated that 3 perceptual factors—problem, constraint, and participation recognition—would determine situational motivation and action (eg, choosing a vaccination brand) [15]. However, we extend the STOPS model by integrating the social support theory and show that official health information (ie, digital intervention) can increase vaccine uptake among individuals with higher situational motivation. Higher problem identification levels inspire people to seek risk information from multiple sources, which can influence their vaccine decision.

**Figure 1:**
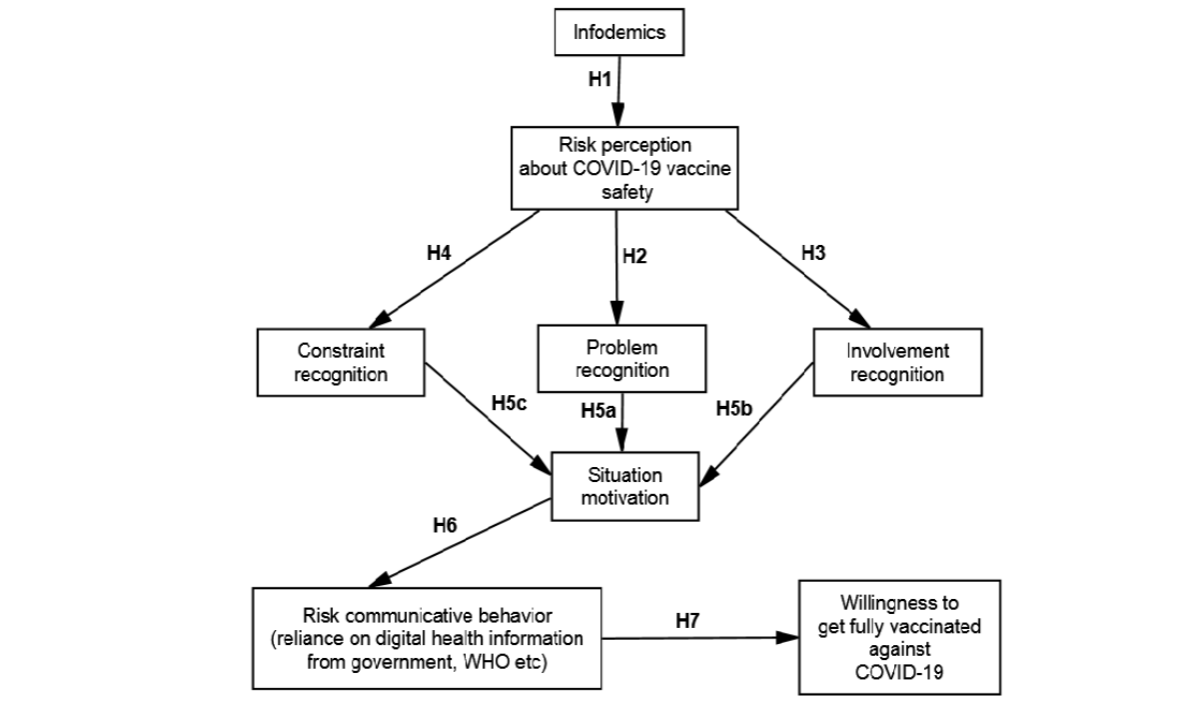
Proposed research model. WHO: World Health Organization.

These perceptual and situational factors will eventually lead to problem-solving–related risk communicative behaviors, namely (1) vigorously seeking information about a particular vaccine brand (eg, risk information seeking) and (2) evaluating all accessible information and choosing those pertinent to the particular vaccine intake issue they face (ie, information forefending). These communicative actions ultimately lead to behaviors related to vaccine intake problems. This research draws upon a behavioral approach to study public decision-making regarding the selection of COVID-19 vaccine uptake. It is closely related to perceptual (trust, safety problem, involvement, and constraint recognition) and situational factors (situational motivation) in problem-solving, risk communication activeness, and ultimately, vaccine uptake intention. The focal merit of the STOPS lies in its ability to forecast the public’s motivation toward problem-solving and problem-solving–related communicative action via some fundamental perceptual and situational factors. This study proposes new contextual antecedents that illuminate the perceptual and situational factors that define the public’s risk communicative actions in solving the vaccine selection problem (Figure 1).

This study extends the implications of the STOPS beyond communicative actions and includes health behaviors related to vaccine intake problems. For instance, infodemics about the risk perception of vaccine side effects will affect perceptual elements and situational motivation. Furthermore, existing studies have rarely linked situational, perceptual, and social aspects. People interact with the digital and real worlds [21]. They receive official information on social health and infodemics. Thus, infodemic management is crucial for vaccination intention. The following sections describe this conceptual paradigm in detail.

#### Individuals’ Exposure to Infodemics and Risk Perception About COVID-19 Vaccine Safety

An infodemic is a form of information overload comprising fake and misinformation disseminated through digital and physical means during disease epidemics [21,29,30]. Infodemics can cause misperceptions and risk-averting behaviors that can endanger one’s health. Infodemics lead to suspicion of health-related preventive recommendations by authorities [29], thus undermining the collective health response. In uncertain circumstances, infodemics negatively influence protective actions [26]. In the current age of digitization and internet, infodemics spread more quickly, consequently increasing the vulnerability to amplified fake information communication [31]. Scholars have noted that infodemics mainly influence people’s beliefs about vaccinations [32]. For example, digital media information consumers have reported several misconceptions about trust in COVID-19 vaccines, including country of origin [16,23], safety concerns [33], privacy concerns [34], fertility concerns, and population controls [27].

Content on several digital websites (eg, blogs) or social media is constantly disseminated by users rather than experts [29]. Along with useful content, research has verified that the interactive nature of digital media generates a powerful forum for disseminating myths, lies, and falsehoods regarding vaccine speculations [35,36]. Studies have also identified that social media facilitates antivaccine propaganda and conspiracy theories [27]. Therefore, it is pertinent to assert that the recent digital media landscape profoundly challenges positive public health responses [21,37]. Unchecked, unverified, and user-generated content may prompt implicit negative beliefs about the COVID-19 vaccine and promote risk perception [29].

According to published research, those who are exposed to infodemics may perceive risk because they are afraid of having severe, adverse COVID-19 vaccine reactions [38]. A sizable portion of the population is concerned about potential side effects that are primarily reported on social media [11]. Therefore, receiving a COVID-19 vaccination is likely to be a risky option for those with a higher risk perception [39]. Extensive analysis revealed that concerns about potential side effects were the most frequently voiced opposition to COVID-19 vaccination [22]. Research has established that social media frequently spreads unverified information about the negative side effects of vaccinations [37,40]. Hence, infodemics spreading via social media may lower people’s perceptions of vaccine safety and enhance their risk perception. These risk perceptions are mainly about the vulnerability to adverse side effects of a particular vaccine brand [38] or country of origin [16], leading to the following hypothesis: infodemics positively influence individuals’ risk perception of COVID-19 vaccine safety due to a lack of trust in the vaccine brand and country of origin (H1).

#### Risk Perception About COVID-19 Vaccine Safety and Problem Recognition

Individuals may perceive an unexpected issue, such as a pandemic, while experiencing a situation. Deviation from an expectation (eg, being healthy) to an observation (such as an illness diagnosis) causes a perception of a troubling situation. Two types of problems have been identified in the literature: perceptual and cognitive problems. Perceptual problem realization occurs when there is a discrepancy between the expected and actual situations. The unexpected onset of the COVID-19 pandemic has caused perceptual issues among the public. Cognitive problem realization occurs when an individual recognizes the absence of a solution to a challenging situation, such as the absence of a COVID-19 vaccine. Cognitive issues require the evaluation of expected actions. Cognitive evaluation assesses various aspects of a problem, including its cause, resolution, and one’s ability to take action to resolve [24,41].

Other perceptual elements influence how people react to the degree of perceived involvement with the issue (awareness of involvement) and the perceived barriers to solving it [28]. Several other parameters affect whether someone *stops* what they are doing “to think about what to do.” In other words, even with a high level of problem recognition, a person may or may not pause when considering what to do [42]. In challenging circumstances such as COVID-19, problem awareness is the leading cause but not the only factor in following communicative and cognitive activities [41]. In this study, we define problem recognition as the belief that being vaccinated against COVID-19 could have negative effects on human health and that the only immediate solution is to obtain the most reputable brand of COVID-19 vaccine currently on the market. This perceptual state can occur when preconscious problem-solving fails. According to the literature, misinformation may cause a perceptual state to be skeptical of the COVID-19 vaccine [43] or infodemics [39]. Previous research has identified several frames of these infodemics deciphering the negative aspects of the COVID-19 vaccine, such as side effects [22], conspiracy beliefs [27], and population control strategies, which could result in a greater extent of perceived problem recognition. Keeping this literature in view, we hypothesize the following: individuals’ risk perception about COVID-19 vaccine safety positively affects their problem recognition of COVID-19 vaccine safety (H2).

#### Risk Perception About COVID-19 Vaccine Safety and Involvement Recognition

The STOPS proposed another perceptual factor included in problem-solving phenomena [24]. The STOPS borrowed this concept from past research that identified involvement as a “degree of relevance.” The STOPS anticipated that a problem’s relevance to a particular product or activity would influence how people would react [15]. Previous research has interpreted the STOPS differently than the participatory idea and labeled it as a nonperceptual factor. As an illustration, scholars previously described involvement as a product characteristic or medium quality [44]. Later, researchers characterized it as a “perception” that people develop in a particular circumstance [15].

In comparison with earlier social psychology theories, the STOPS argued that the intensity to which people connect themselves with a situation is the level of engagement [45,46]. In the context of COVID-19, a person will not initiate action until they notice indicators of an abnormal physical condition—issue recognition—because they are ignorant regarding their vulnerability to contracting COVID-19 (ie, of an actual relationship to the health problem). However, in the case of vaccine safety-related concerns, they feel more involved if they identify that receiving a vaccine of a particular brand, such as Sinopharm, is susceptible to adverse effects. However, if people identify that receiving a vaccine from a specific brand, such as Sinopharm, is susceptible to side effects, they feel more involved in the discussion. Therefore, a more significant risk perception regarding the safety of the COVID-19 vaccine acknowledges individuals’ involvement. Once one identifies the problem, one will only appraise the essentiality of health concerns such as vaccine safety. Therefore, the risk perception regarding vaccine safety outlines the level of involvement. More infodemics-driven risk perceptions can intensify involvement recognition [40,41]. In this regard, we hypothesize the following: individuals’ risk perception of COVID-19 vaccine safety positively affects their involvement in the recognition of COVID-19 vaccine safety (H3).

#### Risk Perception About COVID-19 Vaccine Safety and Constraint Recognition

Unlike some communication principles, recognition has its roots in economics and management science [45]. For example, the perception of individuals about the availability or restrictions of resources outlines decisions, which is a closely related notion of constraint recognition delineated in the STOPS. Previous influential theories extended this concept and applied it to the sociopsychological context [46,47]. For example, the Social Learning Theory proposes a construct of “personal efficacy” to tap into individuals’ resource-driven decision-making [24]. Similarly, the concept of self-efficacy has been presented in self-efficacy theory to tap into one’s perception of their capability to engage in a particular behavior [48]. Aligning with past approaches, the STOPS described constraint recognition as when “people think that there are barriers in a situation that limit their ability to do anything about the circumstance,” and thus, “constrained recognition” occurs [42]. In this study, we adhere to this explanation for constraint recognition. In this scenario, people may evaluate their capacity to deal with a situation, and a constraint assessment process is triggered. For example, myths about COVID-19 vaccine safety can cause them to evaluate the available resources to act in this problematic situation. Therefore, more constraint recognition is expected to increase the risk perception. Previous literature has also advocated that risk awareness directly affects involvement recognition [41,44]. Thus, we hypothesize the following: individuals’ risk perception of COVID-19 vaccine safety positively affects their constraint recognition of COVID-19 vaccine safety (H4).

#### Influence of Problem, Involvement, and Constraint Recognition on Situation Motivation

Past information processing and psychological theories have identified motivational factors that can influence behavioral outcomes in addition to perceptual and cognitive factors [48]. The STOPS added a situational motivation construct, a situation-specific cognitive effort involving the willingness to make problem-solving efforts [46]. A greater extent of situational motivation decreases the apparent inconsistency between anticipated and actual situations [24]. The STOPS tenets of the situational motivation construct are unlike some nonsituational communicative motivations delineated in literature, such as “desire,” “escape,” “relational goals,” or “necessity for social interaction” [45]. The STOPS proposes a more situation-oriented construct to describe individuals’ motivation in problematic situations as a substitute [15]. Instead, the aim-leaning nature of situational motivation is a function of one’s realization (eg, perception) of a problem. Whether a motivational impact exists in a problem-solving scenario and the degree to which it arbitrates the influence of situational perception on information or resource use are both intriguing [47].

This research underpins the concept of situational motivation in problem-solving regarding COVID-19 vaccine safety, which is the decisive factor in outlining communicative behavior. This is consistent with the STOPS, which predicts that situational motivation is influenced by the problem, involvement, and constraint recognition, which constitute the information use model [24,46]. Given that relevant criteria are more cognitive than perceptual, this research draws upon the tenets of the STOPS and argues that their presence will have a separate impact on information use factors. Situational motivation in problem-solving refers to how often a person pauses to consider, is enthralled by, or seeks additional knowledge about a subject [47,48]. If people encounter problematic situations, such as COVID-19 vaccine safety based on their relevance (involvement), vulnerability to side effects (problem recognition), or lack of resources (constraint) for acting to address it, they would have more situation-specific motivation. In this way, a challenging circumstance originates when a person recognizes a problem but feels unable to handle it or lacks the means to minimize the perceived psychological imbalance. Thus, the STOPS argues that owing to the lack of cognitive cues, people start looking at the resources available to them in a problematic situation (eg, COVID-19). The perceived level of disagreement raises the possibility of “stopping to think about what to do,” but it does not define the depth of subsequent consideration alone [49]. For example, a person with fewer perceptual cues regarding vaccine safety can experience a more significant problem because of a lack of trust in a brand. In turn, this motivates a person to receive situation-specific action cues. When people encounter problematic situations, such as risk perception about the COVID-19 vaccine, they may perceive involvement—connection—to the troublesome circumstance that impacts the scope and quality of their communication. By contrast, people are less likely to be motivated by “problems or situations about which they believe they can do little.” According to this literature, we hypothesize the following: individuals’ problem recognition of COVID-19 vaccine safety positively affected their situational motivation with respect to the safety of the COVID-19 vaccine problem (H5a); individuals’ involvement in recognizing COVID-19 vaccine safety positively affected their situational motivation with respect to the safety of the COVID-19 vaccine problem (H5b); and individuals’ constraint recognition of COVID-19 vaccine safety negatively affected their situational motivation with respect to the safety of the COVID-19 vaccine problem (H5c).

#### Communicative Action in Problem Solving

Kim et al [49] developed the notion of a communicative action model. They categorized it into 3 communicative behavioral domains based on two facets—(1) active and (2) passive. These facets decipher the audience’s participation in choosing, conveying, and collecting situation-specific information [11]. The STOPS argues that when individuals pursue solutions to a problem, they mainly imply communicative behavioral dimensions, namely (1) acquisition, (2) transmission, and the selection of information [11]. Each dimension is further divided into 2 categories based on the facet. The information acquisition process involves information seeking (active) and attending (passive).

In comparison, the information selection realm involves information forefending (active) and information permitting (passive). Finally, the information transmission realm includes information forwarding (active) and sharing (passive). In the context of COVID-19 vaccine safety, the term “information forefending” is used to characterize the individual response to the infodemics that trigger the phenomenon of problem-solving [45]. Because of infodemics, a higher degree of perceived risk provokes the mechanism of problem chain identification (eg, problem recognition) that enhances their situational motivation.

For this study, we conceptualized infodemic management through risk communicative behavior as a function of 2 domains related to information acquisition and selection that are mainly available to individuals during COVID-19 in the form of digital interventions made available by official health authorities (eg, WHO) and experts (eg, physicians). In this scenario, individuals search for information, and their decisions about health information rely on how individuals evaluate incoming data for its perceived relevance and avail of it in resolving a given issue [14]. When people actively try to solve a COVID-19 safety problem, they may not accept all the received information; instead, they precisely select the supportive and appropriate ones, for example, the information available to them in a digital advertisement from health authorities and experts [35]. A person would rely on credible data to solve the perceptual and situational gaps created by infodemics regarding vaccine safety.

By contrast, information permitting refers to the degree to which people are willing to accept any information associated with an ongoing challenging circumstance, such as COVID-19 vaccine safety [14]. However, situational motivation is critical in reducing the apparent gap between information needs and relevance [42]. Communicative actions indeed rely on situational motivation; if people feel they need more health information in problem handling, such as COVID-19 vaccine safety, they are inclined to adopt active domains, such as information seeking or forefending. The literature also affirms the role of situation-specific motivation in problem-solving communicative activities [28,42,45]. Previous studies have also found that highly goal-oriented and situation-specific motivations lead to engaging individuals in communication actions in character. Consistent with these tenets, this study proposes the following hypothesis: individuals’ situational motivation positively affects risk-communicative actions in solving the safety problem of the COVID-19 vaccine (H6).

#### Influence of Infodemic Management Through Risk Communicative Actions: Digital Health Intervention by Health Authorities and Experts

Evaluating the health information available to individuals during the COVID-19 pandemic is critical for determining outcomes and positive public health responses [50]. Drawing an analogy with the STOPS, during COVID-19, individuals are more likely to be receptive to information related to COVID-19 vaccine safety. For example, the information forefending and information permitting processes are critical at this stage. In particular, they are associated with the choice of information regarding COVID-19 safety. Moreover, information acquisition about COVID-19 vaccine safety is associated with information seeking and attending, that is, individuals should rely on sources to obtain information about COVID-19 vaccine safety before taking action [14]. In this regard, health authorities’ infodemic management measures have a double-edged effect on understanding problematic circumstances such as COVID-19 susceptibility and providing actionable risk information.

According to the WHO, infodemic management refers to the use of risk communication to deliver factual analysis and actionable cues to the public in an organized manner [1]. This risk communication can aid in the management of infodemics by reducing their adverse effects on public health responses during crises [45]. We argue, in line with the STOPS, that a person who realizes a situation (problem) will seek valuable information to resolve the recognized problem. In this case, infodemics about the safety of the COVID-19 vaccine may reduce health-related behaviors, such as vaccination According to the STOPS, an active problem solver searches for information and processes it to address a problematic issue. A passive one, on the other hand, may just process information or participate in minimal information processing [50]. Consequently, infodemic management is critical to bolster positive health responses by providing targeted risk information about the identified problem. Thus, desired active communicative behaviors can be ensured by providing timely and factual analysis based on information to address concerns and questions about COVID-19 vaccine safety. In this regard, the social support theory also advocates information support’s role in better understanding the public, mainly when they require information [50,51].

Previous research on risk communication and COVID-19 has found that health expert advice (eg, physicians) and official sources are among the most credible and influential sources for determining public responses, such as willingness to get a vaccination [14,50]. Therefore, the public can benefit from infodemic management (dissemination of risk information) from these sources. Some studies have also noted that information from medical professionals and official authorities could elicit a favorable public reaction [39]. Therefore, the official authorities’ infodemic management through risk communication strategies involving health experts can build more resilience to counter infodemics, and it is hypothesized as follows: infodemic management through communicative actions in solving the COVID-19 vaccine safety problem positively affects the WFVC (H7).

### Research Design

This study used a cross-sectional research design vis-à-vis a web-based survey to examine the adversities (risk perception about COVID-19 vaccine safety) associated with infodemics and the underlying mechanisms of perceptual, situational, and motivational variables, resulting in risk communication behavior that leads to WFVC. Owing to this study’s purpose, internet users possibly exposed to COVID-19 vaccine–related content were the target population. The criteria for inclusion of the respondents were (1) age ≥18 years and (2) internet users exposed to information about COVID-19 vaccine–related infodemics. A nationally representative sample of 1946 internet users across Pakistan was collected using a web-based questionnaire administered through Google Forms. The web link to the questionnaire was posted on numerous social media platforms. Web-based data collection suited the aims and nature of this research and the desire to approach real-time social media users who are susceptible to infodemics. A filter question was asked regarding exposure to infodemics. Participants voluntarily participated in this research after completing an informed consent form and reading ethical permissions. Responses were received over 3 months, from May 2022 to July 2022. Pakistan had approximately 89.1 million active internet users in January 2022. This study undertook a 2-fold strategy to confirm sample generalization. First, this research used a G-Power analysis, which confirmed that a sample size greater than 1800 is appropriate, with an effect size (f) of 0.4892 and power of 0.90 (*P*=.001). Second, Krejcie and Morgan’s sample size determination formula also authenticated that a sample size of 1537 can exhibit adequate generalization for a population of less than 100 million with a confidence level of 2.5%. Therefore, a minimum of 1800 adult internet users would have been required to execute this research. However, this research used covariance structural equational modeling that requires data to meet the normality assumption. Therefore, approximately 15%-20% more data were collected as a caution to avoid generalizability issues in the case of outlier case deletion to meet data normality assumptions. Translational and content validities were obtained from 10 experts and academicians. The questionnaire and construct definitions were sent out to them to read, and they were asked to rate their suitability on a 4-point Likert scale. After obtaining their responses, the content validity rating was calculated and found to be within the suggested threshold of 0.66. However, language adjustments were incorporated according to feedback. The demographic analysis demonstrated a plurality of respondent characteristics; among the 1946 respondents, 1067 (54.83%) were men, and 879 (45.17%) were women. Of the 1946 respondents, a plurality of respondents, 868 (44.6%) were aged between 18 and 30. Of the 1946 respondents, the number of unmarried respondents were 1327 (68.19%), 588 (30.22%) were married, and 31 (1.59%) were divorced. A total of 823 (42.29%) participants had a college degree. The demographic characteristics of the participants are shown in Table 1.

**Table 1:** Demographic characteristics (N=1946).

Demographic		Frequency, n (%)
**Gender**		
	Man	1067 (54.8)
	Woman	879 (45.2)
**Marital status**		
	Unmarried	1327 (68.2)
	Married	588 (30.2)
	Divorced	31 (1.6)
**Area**		
	Urban	1639 (84.2)
	Rural	307 (15.8)
**Age range (years)**		
	18-30	868 (44.6)
	31-44	527 (27.1)
	45-59	394 (20.2)
	≥60	157 (8.1)
**Education level**		
	High school certificate	619 (31.8)
	College diploma	823 (42.3)
	University degree	504 (25.9)

### Ethical Considerations

The study was conducted in accordance with the guidelines of the Declaration of Helsinki and approved by the Institutional Research Ethical Committee of Centre for Media and Communication Studies, University of Gujrat, Gujrat 50,700, Pakistan (Ref.No.UOG/CMCS/2022/395B). This research also followed the standards for reporting web-based surveys—CHERRIES (The Checklist for Reporting Results of Internet E-Surveys) checklist—and informed consent was obtained from all participants involved in the study. All data were collected anonymously and kept confidential. There was no remuneration or compensation offered to the respondents. Informed consent was obtained from all participants involved in the study.

### Measurement

The variable of infodemics was measured using 5 items adapted from literature. The items were as follows: (1) “COVID-19 vaccine development did not involve valid safety testing,” (2) “COVID-19 vaccine contains dangerous nanoparticles that will affect human health,” (3) “COVID-19 vaccine is a Population Control Mechanism,” (4) “The microchip can be implanted in my body through COVID-19 Vaccine,” and (5) “The COVID-19 vaccine negatively affects human health.” In this study, risk perception about COVID-19 vaccines was measured using 4 items adapted from the literature [52]. Problem, involvement, and constraint recognition were measured using 4 items for each variable, adapted from the literature [24,25]. Situational motivation was measured using 3 items adapted from the literature [24,25]. Risk communicative behavior is a second-order construct, including information seeking, forfending, attending, and permitting. Each dimension was measured using 2 items suggested in the literature [24,25]. WFVC was measured using 3 items adapted from the literature [53]. All items involved in this research were measured on a 5-point Likert scale “(5=strongly agree, 4=agree, 3=neutral, 2=disagree, and 1=strongly disagree).”

## Results

### Overview

SPSS software (version 22.0; IBM Corp) was initially used to perform several statistical tests, including (1) data normality, (2) outlier visualization, (3) variance inflation, and (4) Pearson test for correlation. The findings revealed satisfactory normality of the data after removing 85 outliers. Furthermore, the variance inflation tests revealed no threatening issue regarding multicollinearity in the data; all items were reported to be far below the cut-off value of 10. The results of the bivariate correlations are presented in Table 2, and the variables are correlated, as theorized in this study.

**Table 2:** Correlations.

Variables	Mean (SD)	α	Infodemics	RPCV^a^	PR^b^	IR^c^	CR^d^	SM^e^	RCB^f^	WFVC^g^
Infodemics	3.70 (1.23)	.876	1	—^h^	—	—	—	—	—	—
RPCV	4.39 (0.889)	.887	0.18	1	—	—	—	—	—	—
PR	3.99 (0.747)	.816	0.37	0.13	1	—	—	—	—	—
IR	3.68 (0.718)	.810	0.57	0.47	0.52	1	—	—	—	—
CR	3.29 (0.724)	.881	−0.11	0.29	0.40	0.14	1	—	—	—
SM	4.37 (0.955)	.795	0.35	0.26	0.31	0.54	0.17	1	—	—
RCB	4.16 (0.887)	.896	0.26	0.12	0.34	0.28	0.20	0.27	1	—
WFVC	3.89 (0.681)	.843	0.29	0.26	0.10	0.38	0.11	0.36	0.53	1

^a^RPCV: risk perception about COVID-19 vaccine safety.

^b^PR: problem recognition.

^c^IR: involvement recognition.

^d^CR: constraint recognition*.*

^e^SM: situational motivation.

^f^RCB: risk communicative behavior.

^g^WFVC: willingness to get fully vaccinated against COVID-19.

^h^Not applicable.

### Confirmatory Factor Analysis

After the descriptive analysis, this study conducted a confirmatory factor analysis using structural equation modeling (SEM) techniques on Amos 23.0 (IBM Corp). SEM is a better method for validating the internal reliability, validity, and goodness-of-fit model. The confirmatory factor analysis measurement results exhibited an excellent fit model (Table 3). The recommended acceptable values of the goodness-of-fit index, Turkey-Lewis Index, incremental fit index, and comparative fit index are between 0.90 and 1. Similarly, a root mean square error of approximation value of below 0.60 is considered satisfactory, and the chi-square per *df* must be between 1 and 5. The values presented in Table 3 suggest acceptable values for the chi-square per *df*, and absolute and incremental indices (eg, comparative fit index and Turkey-Lewis Index) aligned with these recommended values.

Subsequently, the convergent validity and internal reliability were investigated based on the recommended threshold values of the composite reliability (above 0.80) and the average variance extracted (above 0.50).

**Figure 2:**
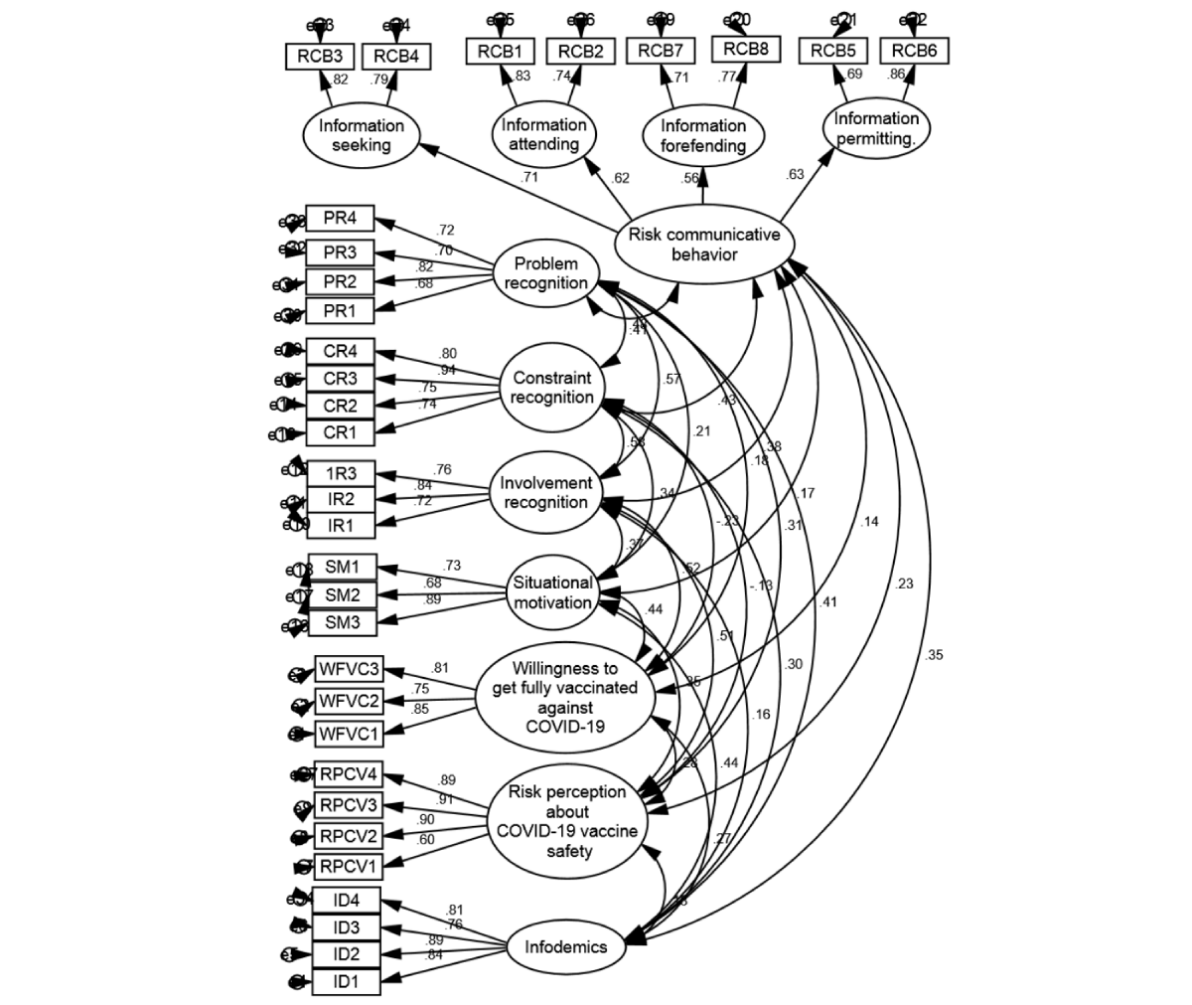
Measurement model. CR: constraint recognition; ID: infodemics; IR: involvement recognition; PR: problem recognition; RCB: risk communicative behavior; RPCV: risk perception about COVID-19 vaccine safety; SM: situational motivation; WFVC: willingness to get fully vaccinated against COVID-19.

The Fornell and Larcker technique was used to estimate the discriminant validity across all research constructs. The findings demonstrated that discriminant validity was established because the associations between variables and their original variables were stronger than their shared associations with other variables in the measurement model (Table 4). Before performing inferential statistics, a structural model was assessed for goodness-of-fit model indices, and the results were satisfactory (Table 3). Item loadings are accessible in Figure 2 and Table 5.

**Table 3:** Confirmatory factor analysis.

Measurement models	Chi-square (*df*)	GFI^a^	TLI^b^	IFI^c^	CFI^d^	RMSEA^e^
Measurement model	2379 (3.56)	0.97	0.93	0.93	0.96	0.042
Structural model	1822 (2.67)	0.93	0.92	0.91	0.93	0.045

^a^GFI: goodness-of-fit index.

^b^TLI: Turkey-Lewis Index.

^c^CFI: comparative fit index.

^d^IFI: incremental fit index.

^e^RMSEA: root mean square error of approximation.

**Table 4:** Validity.

Variables	CR^a^	AVE^b^	Infodemics	RPCV^c^	PR^d^	IR^e^	CR	SM^f^	RCB^g^	WFVC^h^
Infodemics	0.895	0.682	(0.825)^i^	—^j^	—	—	—	—	—	—
RPCV	0.902	0.697	0.18	(0.834)	—	—	—	—	—	—
PR	0.821	0.535	0.41	0.31	(0.731)	—	—	—	—	—
IR	0.817	0.601	0.16	0.51	0.57	(0.775)	—	—	—	—
CR	0.884	0.658	0.30	−0.13	0.48	0.58	(0.811)	—	—	—
SM	0.813	0.595	0.44	0.35	0.21	0.31	0.19	(0.771)	—	—
RCB	0.923	0.601	0.35	0.23	0.41	0.38	0.34	0.17	(0.775)	—
WFVC	0.845	0.647	0.27	0.28	0.18	0.52	−0.23	0.44	0.23	(0.804)

^a^CR: constraint recognition*.*

^b^AVE: average variance extracted.

^c^RPCV: risk perception about COVID-19 vaccine safety.

^d^PR: problem recognition.

^e^IR: involvement recognition.

^f^SM: situational motivation.

^g^RCB: risk communicative behavior.

^h^WFVC: willingness to get fully vaccinated against COVID-19.

^i^Values in parentheses represents Square root of Average Variance Extracted.

^j^Not applicable.

**Table 5:** Standardized loadings.

Variable		Item loadings
**ID^a^**		
	ID1	0.84
	ID2	0.89
	ID3	0.76
	ID4	0.81
	ID5	0.54^b^
**RPCV^c^**		
	RPCV1	0.60
	RPCV2	0.90
	RPCV3	0.91
	RPCV4	0.89
**PR^d^**		
	PR1	0.68
	PR2	0.82
	PR3	0.70
	PR4	0.72
**IR^e^**		
	IR1	0.72
	IR2	0.84
	IR3	0.76
	IR4	0.51^f^
**CR^g^**		
	CR1	0.74
	CR2	0.75
	CR3	0.94
	CR4	0.80
**SM^h^**		
	SM1	0.73
	SM2	0.68
	SM3	0.89
**RCB^i^**		
	RCB1 information attending (dimension)	0.80
	RCB2	0.74
	RCB3 information seeking (dimension)	0.83
	RCB4	0.79
	RCB5 information permitting (dimension)	0.69
	RCB6	0.86
	RCB7 information forefending (dimension)	0.71
	RCB8	0.77
**WFVC^j^**		
	WFVC1^j^	0.85
	WFVC2	0.75
	WFVC3	0.81

^a^ID: infodemics.

^b^Removed items.

^c^RPCV: risk perception about COVID-19 vaccine safety.

^d^PR: problem recognition.

^e^IR: involvement recognition.

^f^deleted item.

^g^CR: constraint recognition.

^h^SM: situational motivation.

^i^RCB: risk communicative behavior.

^j^WFVC: willingness to get fully vaccinated against COVID-19.

### Hypothesis Testing

This study proposed 7 hypotheses and used SEM path analysis to test these assumptions. H1 predicted the influence of infodemics on the RPCV. The SEM results revealed a significantly positive (β=.43; *P*=.001) influence of infodemics on RPCV; therefore, H1 was supported. H2 postulated a positive influence of individuals’ RPCV on problem recognition. The path analysis revealed a positive and significant coefficient (β=.46; *P*=001), which supported H2. The third hypothesis (H3) assumed a positive influence of individuals’ RPCV on involvement recognition. The path analysis showed a positive and significant coefficient (β=.51; *P*=.001), which implies that H3 is supported. Furthermore, H4 assumed a positive influence of individuals’ RPCV on constraint recognition; the path analysis showed a positive and significant coefficient (β=.34; *P*=.04), supporting H4.

**Figure 3:**
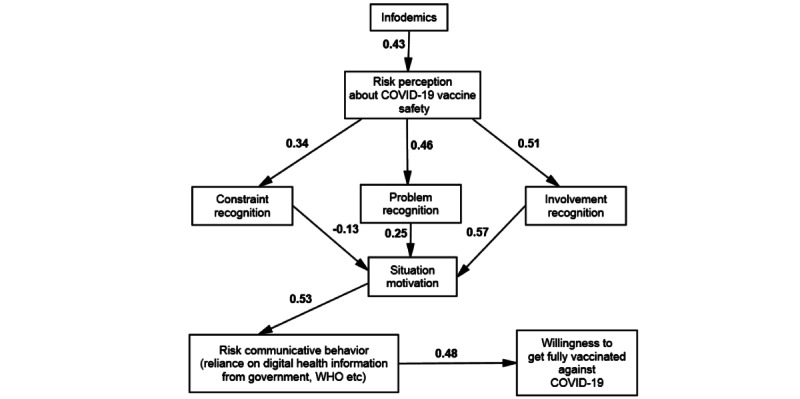
Structural model.

The results of the SEM path analysis also tested the influence of the (H5a) problem (β=.25; *P*=.01), (H5b) involvement (β=.57; *P*=.001), and (H5c) constraint recognition (β=−.13; *P*=.001) on situational motivation in solving vaccine uptake. Thus, H5a, 5b, and 5c were supported. H6 predicted a positive influence of situational motivation on risk communication behavior, which was also supported and found significant (β=.53; *P*=.001; *R^2^*=0.40). Finally, the direct influence of risk communication behavior was positive and significant (β=.48; *P*=.001; *R^2^*=0.46) on WFVC, supporting H7 (Figure 3 and Table 6).

**Table 6:** Hypothesis testing.

Direct influences	β	*P* value	*t* value	Hypothesis
Infodemics → RPCV^a^	.43	.01	11.18	H1 supported
RPCV → problem recognition	.46	.001	8.74	H2 supported
RPCV → involvement recognition	.51	.001	10.83	H3 supported
RPCV → constraint recognition	.34	.04	4.13	H4 supported
Problem recognition → situational motivation	.25	.001	8.72	H5a supported
Involvement recognition → situational motivation	.57	.001	6.17	H5b supported
Constraint recognition → situational motivation	−.13	.001	7.21	H5c supported
Situational motivation → risk communicative behavior	.53	.001	8.21	H6 supported
Risk communicative behavior → WFVC^b^	.48	.001	9.36	H7 supported

^a^RPCV: risk perception about COVID-19 vaccine safety.

^b^WFVC: willingness to get fully vaccinated against COVID-19.

## Discussion

### Principal Findings

The purpose of this study was multifold: (1) to explain and predict the influence of infodemics in the promotion of risk perception about COVID-19 vaccine safety; (2) to understand the role of risk perception in outlining the individuals’ perceptual, cognitive, and situational engagement in adopting communicative behavior in problem-solving; and (3) to use the health information seeking approach to determine the efficacy of the information management strategies used by the health authorities. To meet these purposes, this study applied the STOPS to measure the outcome variable of WFVC and verify the role of communicative actions in the problem chain identification effect. This study proposed several hypotheses to test the relationship between the constructs involved in this problem chain identification and its outcomes. H1 suggests that infodemics predict risk perception, as validated by an empirical study. H2, H3, and H4 posited that risk perception, which is a function of infodemics, leads to realization of the problem, involvement, and constraint evaluation. Our findings supported H2, H3, and H4 and revealed that the prevailing threat of risk perception hinders vaccination. This is consistent with previous studies that support a similar threat to public hesitancy due to myths disseminated by social media [29,37]. The result of H5 also verified the STOPS’s notion that if a person realizes a problematic situation, it enhances their motivation to obtain domain-specific information. These results align with those of prior studies that verified that the problem-recognition chain influences situational motivation [42,48].

Furthermore, H6 led to a better understanding of individuals’ information behaviors concerning issues related to COVID-19. It has contributed to the development of public strategies to facilitate others adopting a favorable position on readiness to be fully vaccinated. Previous studies have also found that situational motivation intensifies communicative behavior [45,48]. In the context of COVID-19 vaccination, these results are noteworthy and valuable for health authorities managing infodemics. The results for H6 revealed that the negative influences of risk perception and infodemics, such as vaccine hesitancy, are not the ultimate consequence [9,10,32]. However, regardless of this negativity, this study has demonstrated that people also implement a problem-solving approach. For example, H6 demonstrated that the development of situational motivation improves communicative actions, including information seeking. Therefore, this research made a substantial contribution by validating the idea of information behaviors commonly known as communicative action in problem-solving. This is more comprehensive and all-encompassing compared with the previous notion of negative influences. In the past, many studies on health communication concentrated on information acquisition and selection [41,48].

This study identified 4 critical communicative behaviors for managing infodemics. These dimensions are primarily related to information acquisition and selection. According to our research findings, the risk-communicative actions of individuals are connected to WFVC. Previous studies have also identified the relationship of communicative action with behavioral outcomes and are on par with the findings of this research [14,42]. Risk communication actions are primarily associated with risk management such as infodemics [39]. The results showed that respondents who came into contact with the information (digital interventions) provided by official authorities or health experts were encouraged to obtain additional information. Consequently, the current digital media landscape influences health outcomes [21,37]. Thus, the results suggest that the desire to obtain situation-specific information corresponds to encouraging public health responses [41]. The findings also indicate that people tend to identify the connections between one’s WFVC issue and other related difficulties, such as vaccine safety. Overall, this study suggests that as individuals’ level of concern increases because of infodemics, a phenomenon known as the “problem chain recognition effect” starts which corresponds to an increase in situational motivation [24,46]. Increased situational motivation enhances reliance on active information seeking. Therefore, reliable digital health interventions (eg, physicians) and government authorities can significantly determine the willingness to be fully vaccinated against COVID-19 [14,50]. However, infodemic management with situation-specific information is an important factor in improving public response. In summary, identifying the spread of infodemics among the public will be an excellent strategy for strengthening the efficacy of health campaigns.

### Theoretical Implications

This research extended the theoretical explanation of the STOPS by integrating notions of social support theory and thus presented a more significant number of theoretical assumptions. Furthermore, the study applied a novel context related to health concerns that helped explain the central communicative role in reducing the adverse effects of infodemics. The results robustly advance the implications of the STOPS in health-related problem-solving. The STOPS offers relative advantages when describing and explaining the more complicated phenomenon of communicative action (eg, members of a public choosing which information to seek). However, this research advances the STOPS by using diverse approaches to describe the communicative environment during COVID-19. As COVID-19 vaccination is a solution, misinformation based on rumors, myths, and conspiracies about the vaccine brand or country of origin is a problem [10,26]. This study used the STOPS because of its situational attributes and theoretical strength. It integrates it with notions of social support theory to develop a more comprehensive problem-solving approach [48,51].

In line with the positive psychology approach, we argue that infodemics not only are a communicative environment during COVID-19 but also include infodemic management through several risk communication strategies [39]. Therefore, the infodemic management concept was reoperationalized in this study to propose a model that can provide a more reasonable solution for resolving COVID-19 safety concerns. Consequently, we explored the new theoretical implications of the STOPS and its substantial contributions to experts in the field of communication (discussed in the following section). This study shows that the STOPS is a valuable tool for health communication and supports its relevance as a theoretical framework. Understanding health issues is a prerequisite to developing positive attitudes, intentions, and behaviors. This study sheds light on how people learn about and process health-related information. As they are aware of the issues, limitations, and stakes in finding solutions, these people are motivated to take action to remedy the current dire lack of willingness to take vaccine shots. Thus, this theory becomes handy and supports researchers who are determined to find the theoretical process by which individuals become motivated concerning COVID-19 vaccine safety issues. It prepares a blueprint for strategic health communication campaigns to raise individuals’ levels of responsiveness to COVID-19 vaccine hesitancy. To reiterate, the identification of infodemics among the public and infodemic management vis-à-vis reliable digital interventions is an excellent strategy to combat resistance against vaccination programs and to strengthen the efficacy of health campaigns*.* This study should be repeated in various health-related settings in the future.

### Practical Implications

The communicative approach in research has received little public attention. Using the STOPS in the infodemics and its management domain, identification of the problem chain effect, and digital interventions will help health campaigns on vaccine safety. Health communicators must frequently promote public awareness to rectify issues such as COVID-19 vaccine hesitancy owing to higher levels of immediate importance. This research will encourage health communicators to design and implement more effective communication initiatives to manage infodemics, especially when they understand the effect of problem chain recognition. Targeted campaigns can be launched to address the targeted problems, resulting in more positive public engagement.

Health communicators can create various communication agendas and strategies by reaching out to the public to clarify misinformation. However, identifying more prominent references and their sources is critical to obtain efficient results from health campaigns. The problem-identification approach can help address specific requirements and features of the selected health concern (eg, how to reduce risk perception) and organize campaigns accordingly. The results provide examples of how campaign objectives can be defined using STOPS tenets by performing a simple evaluation matrix before designing health campaigns.

### Limitations and Future Research

First, although the results match the predictions, using nonrepresentative national samples is not ideal. To generalize our findings, future research must use nationally representative samples from other countries and other immunization issues such as polio. Second, multiple vaccination-related questions in one survey increased the participant similarity. Therefore, reducing response bias may require further research. This study attempted to avoid this, but its goal was to identify the problem chain recognition effect that may have overlooked concerns. Third, future studies could simulate the impact of chain recognition in real life using quasi-experimental methods to provide a more sophisticated causal effect. Finally, this study did not highlight the referent criterion, which future studies must measure to assess the subjective judgment rules people use to solve the COVID-19 vaccination problem.

### Conclusions

The understudied mechanisms of perceptual, situational, and motivational factors that may have fostered negative perceptions of COVID-19 vaccine safety were investigated in this study. This study revealed how the STOPS could support WFVC in active public participation in information-seeking patterns. Therefore, we conclude that the likelihood of managing infodemics using situational context through exposure to relevant information could improve one’s knowledge of forfending and selection, which can lead to robust WFVC. Therefore, more situation-specific information about the underpinning problem (ie, the selection of an appropriate vaccine) can be made accessible through several official digital sources. This would lead to a more active public health response. In summary, health communicators can become more strategic as they comprehend the theoretical justification and conclusions of the STOPS. In light of our results, health communication campaigns can use the STOPS approach to obtain positive results. They must consider the elements of information-seeking and situational factors that have been overlooked in the past. The communicative cues suggested a direct link with the COVID-19 vaccine shot uptake. Therefore, high-quality information support (eg, using experts) can effectively improve positive public health responses.

## Abbreviations

CHERRIES: The Checklist for Reporting Results of Internet E-Surveys
SEM: structural equation modeling
STOPS: Situational Theory of Problem Solving
WFVC: willingness to get fully vaccinated against COVID-19
WHO: World Health Organization

## Data Availability

All data and materials can be obtained from the corresponding author upon reasonable request.
